# Granulocytic sarcoma of the breast in acute myeloid leukemia: Two case reports

**DOI:** 10.3892/ol.2013.1687

**Published:** 2013-11-19

**Authors:** JIANFEI FU, JIANSHENG LUO

**Affiliations:** Department of Oncology, Jinhua Central Hospital, Jinhua, Zhejiang 321000, P.R. China

**Keywords:** breast, granulocytic sarcoma, myeloid leukemia

## Abstract

Granulocytic sarcoma (GS) of the breast is extremely rare in patients with acute myeloid leukemia (AML) and therefore, is often misdiagnosed as lymphoma or other benign tumors. The current report presents two cases of GS of the breast, of which, one was considered to be a fibroma, as observed by fine-needle aspiration, and the other was misdiagnosed as lymphoma by frozen section. Previous literature that described the clinical and pathological characteristics, treatments and prognosis of GS of the breast in AML were reviewed. In addition to the treatment of mastectomy with/without radiotherapy, lumpectomy may also be received as a good treatment plan.

## Introduction

Granulocytic sarcoma (GS) is a solid tumor containing myeloid leukemia cells that appears in a variety of locations, including the central nervous system, bone and soft tissue. To the best of our knowledge, GS invading the breast is rare (1) and may present at the onset of acute myeloid leukemia (AML) or extramedullary relapse following bone marrow transplantation. No characteristic appearances of GS of the breast have been identified by radiography or ultrasonography. In addition to its rarity, GS of the breast is easy to misdiagnose as other benign tumors, lymphoma or sarcoma. Currently, the only method to confirm the diagnosis of GS of the breast is by pathological examination combined with immunohistochemistry. Doctors must take GS of the breast into consideration when the diagnosis is confusing. The standard therapeutic approach for GS of the breast remains undefined; therefore, doctors face a dilemma in making a clinical decision. The current report presents two cases of GS of the breast and a review of the literature. This study was approved by the Ethics Committee of Jinhua Central Hospital and was performed according to the Declaration of Helsinki. Written informed consent was obtained from each patient’s family.

## Case reports

### Case 1

A 59-year-old female presented on June 25, 2008, with a painless mass in the left breast that had been present for one week and a fever with a temperature of 37.0–37.7ºC. The patient exhibited no other symptoms or relevant past or family histories. A physical examination revealed a mobile mass in the left breast measuring 1.8×1.2 cm, with no palpable auxiliary lymph nodes.

Ultrasound revealed a solid and hypoechoic mass measuring 1.7×0.9 cm, which had a clear boundary with hypervascular flow, and the mammography showed an equidensity mass with no areas of calcification. The chest radiography revealed no abnormal observations, and abdominal ultrasound revealed no suspected lesions in the liver or spleen. The complete blood count showed a white blood cell (WBC) count of 2.9×10^9^/l, a neutrocyte count of 0.2×10^9^/l, hemoglobin levels of 109 g/l and a platelet (PLT) count of 104×10^9^/l.

**Figure 1:**
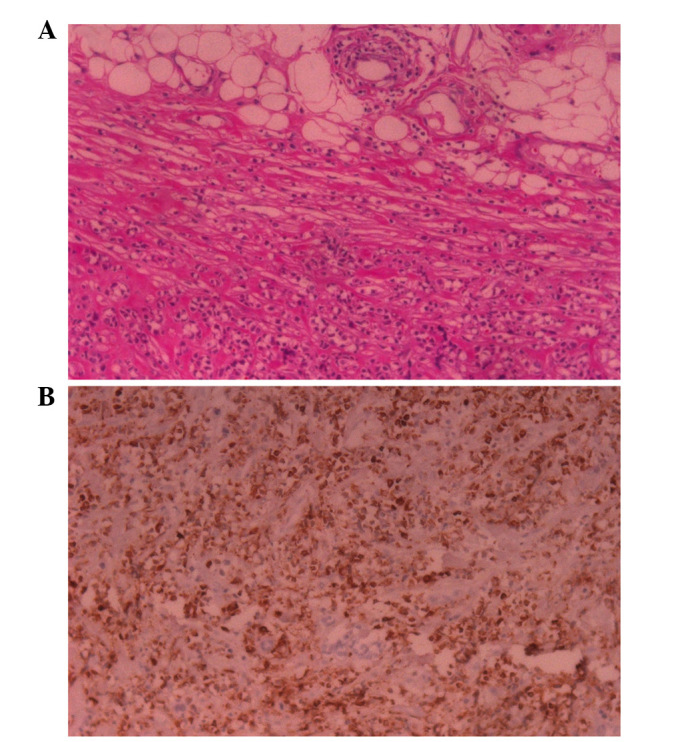
(A) In case 1, the normal breast structure was destroyed. At the edge of tumor, tumor cells were compressed by massive fibrous tissue into a streamline alignment. (B) Immunohistochemistry stain showing that MPO was markedly positive in the cell plasmid. (Hematoxylin and eosin staining; magnification, ×200).

Since the mass in the patient’s breast was considered to be a benign tumor, a lumpectomy had to be performed under local infiltration anesthesia. The patient underwent the lumpectomy on June 10, 2008, despite a relatively low WBC count. According to the frozen section, the patient was diagnosed with breast mucosa-associated B-cell lymphoma. Post-operative pathology revealed that the nuclei of the tumor cells were evidently different in size and shape and scattered among the massive fibrous tissue. At the edge of the tumor, the tumor cells exhibited a streamline alignment (Fig. 1A) and mitotic figures were easily identified. The immunohistochemical analysis showed that markers of myeloperoxidase (MPO) and CD45RA were positive and those for carcinoembryonic antigen (CEA), L26 and CD3 were negative, while CD45RO was weakly positive (Fig. 1B). The patient was confirmed with AML-M4E0 by bone marrow aspiration, and the final diagnosis was GS of the breast.

The patient received inducted chemotherapy with the MA regimen (i.v. injection of 6 mg/m^2^ mitoxantrone days 1–3 plus i.v. injection of 100 mg/m^2^ cytarabine day 1–3, every 3 weeks) and achieved complete remission. The patient subsequently received consolidation chemotherapy. To date, the patient has received follow-up for 4 years and no relapse has occurred.

### Case 2

A 37-year-old female was diagnosed with AML-M6 by bone marrow aspiration and biopsy in April, 2004. The patient achieved complete remission following inducted chemotherapy with retinoic acid (45 mg/m^2^, days 1–28). Following a total of 8 cycles of consolidation chemotherapy with the MA regimen, the patient underwent allogeneic bone marrow transplantation in January, 2006.

In November 2006, the patient was admitted to the Department of Oncology (Jinhua Central Hospital, Jinhua, China) due to a painless palpable mass in the left breast that had been present for 10 days. The patient exhibited no other symptoms and had no relevant previous or family histories. A physical examination revealed a mass measuring 2.0×1.5 cm in the lower inner quadrant of the left breast. Ultrasound revealed a solid and hypoechoic mass, and fine-needle aspiration (FNA) identified the mass as a fibroma. No suspicious lesions were located in the patient’s lungs by chest radiography or in the liver by ultrasound.

**Figure 2:**
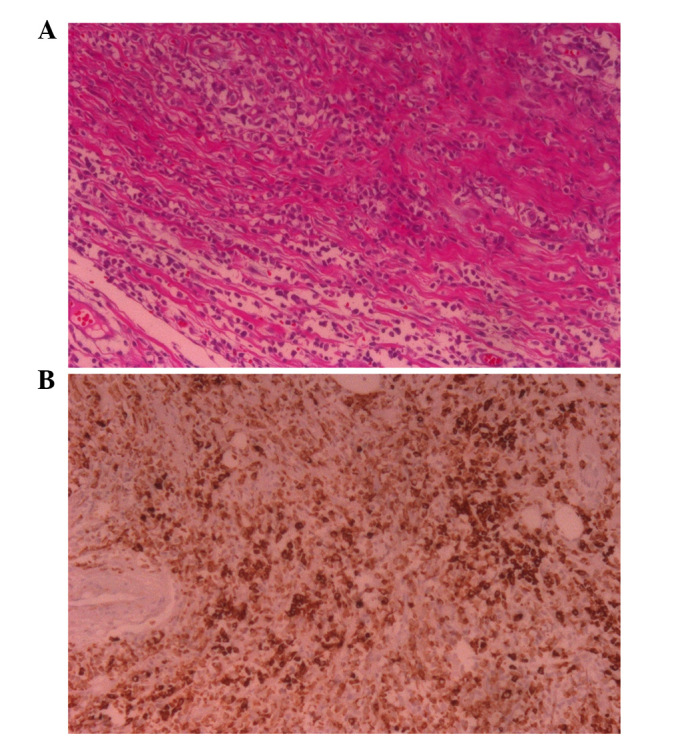
(A) In case 2, mesenchymal tissues were packed into the tumor cells, arranged into a line and the fatty tissue was infiltrated at the edge of the tumor. (B) Immunohistochemistry stain showing that MPO was markedly positive in the cell plasmid. (Hematoxylin and eosin staining; magnification, ×200).

The complete blood count showed a WBC count of 2.9×10^9^/l with 53.4% neutrocytes, a red blood cell count of 1.94×10^12^/l with 72.0% HCT and a PLT count of 27×10^9^/l. Since the mass was diagnosed as a benign tumor by FNA, the patient underwent a lumpectomy under local infiltration anesthesia, despite having a low PLT count. The tumor was removed in November, 2006, and the pathological results revealed that the structure of the breast, which had been invaded by middle-sized diffuse lymphoid cells and fat tissue, was destroyed (Fig. 2A). Immunohistochemical analysis revealed that the markers of MPO and LCA were positive and that those for CD68, CD79a and L26 were negative (Fig. 2B). Following confirmation by histopathology, the patient was diagnosed with GS of the breast. The tests for liver function, performed in February, 2007, demonstrated that the levels for alanine aminotranferase, aspartate aminotransferase, total bilirubin, direct bilirubin and r-glutamyltransfarase were 97 IU/l, 186 IU/l, 90.7 μmol/l, 40.8 μmol/l and 1,064 U/l, respectively, with normal reference values of 0–40 IU/l, 0–40 IU/l, 2–25 μmol/l, 0–8 μmol/l and 0–50 U/l, respectively. The level of the immunoglobulin IgG protein subtype was 4,240 mg/ml (normal reference value, 723–1,685 mg/ml). The patient was diagnosed with graft-versus-host-disease (GVHD) and received immunosuppression therapy to protect the liver. However, the effect of the treatment was unsatisfactory and the patient succumbed to fungal pneumonia in June, 2007, without having received systemic chemotherapy or a second bone marrow transplantation. The relapse of the GS in the patient’s breasts was not identified prior to mortality.

## Discussion

The prevalence rate of GS is ~8% of all AML cases (1). GS may occur simultaneously with leukemia or as the initial symptom of recurrence following bone marrow transplantation in patients with AML. The mechanism of GS remains undefined and no definite associations between GS and bone marrow transplantation have been identified. The French-American-British classification subtypes of AML, M4 and M5, and a high WBC count may be predictors of GS (2). However, in the current report, case 1 was diagnosed with M4 and case 2 was diagnosed with M6, and a high WBC count was not identified in either case, while a low WBC count was identified in case 1. The results are inconsistent with the literature (2) and therefore, further study is required to detect predictors of GS. The mechanism of extramedullary relapse following allogeneic bone marrow transplantation remains unclear. There have been numerous cases of extramedullary relapse and normal donor cells in the bone marrow and it is possible that extramedullary relapse results from the graft-versus-leukemia (GVL) effect (3,4). The graft induces the remaining leukemia stem cells in the marrow to release into extramedullary sites. The patient in case 2 was diagnosed with GVHD, and a previous study demonstrated that GVHD and GVL have the same mechanism (5). However, no reports have analyzed the correlation between GVHD and the extramedullary relapse of AML.

For patients with GS of the breast, the isolated nodule, multiple nodules or mass may be observed in the unilateral or bilateral breasts of patients of any age, even with a diffuse lesion. Patients do not present specific signs, including nipple inversion or discharge (6) and do not have a family history of breast cancer. The two cases in this report presented with unilateral isolated nodules. The ultrasound observations for GS of the breast are absent of characteristic appearances and often present as a hypoechoic and well-defined mass, with color flow imaging and without areas of calcification (7). Few case reports have previously described the mammographic appearance of GS of the breast (8,9). GS has been commonly described as a non-calcified mass in the breast. In case 1, the mammogram also presented a non-calcified, irregular mass, consistent with studies in the literature (10). The absence of characteristics in the imaging for GS results in the difficulty of differentiating GS from other breast diseases. Therefore, pathological analysis is necessary in order to confirm the diagnosis of GS. However, without sufficient preparation, due to the rarity of GS of the breast, the condition is usually misdiagnosed as a benign tumor or primary carcinoma of the breast by FNA, as was evident in the two cases of the current study. A biopsy is the only method to confirm the final diagnosis of GS, however, hematoxylin and eosin (H&E) staining may reveal various morphological changes, resulting in the common misdiagnosis of GS as lymphoma or sarcoma (9). H&E staining may reveal tumor cells that vary in size and with evident nuclear atypia (11). At the edge of the tumor, the vast mesenchymal tissue compresses the tumor cells into a streamline alignment. The immunohistochemical detection of MPO-positive cells is useful in order to confirm the final diagnosis, and primary breast carcinoma may be ruled out by the detection of cytokeratin-negative cells. B cell and T cell markers may be useful as further indicators for ruling out the diagnosis of lymphoma. By combining the history of AML with observations made in a bone marrow aspiration or biopsy, GS of the breast may be confirmed.

The therapeutic approaches for GS of the breast remain unconfirmed and there have been controversial opinions with regard to the local treatment of the breast. Mastectomy or lumpectomy with or without radiotherapy may be acceptable as treatments, and radiotherapy has been recommended in specific previous studies, however, the role of surgery remains unclear (12). The two cases presented in the current report were treated with lumpectomy alone and the results of local recurrence in the breast were satisfactory, as no relapse was identified in either of the patients. A lumpectomy may be an alternative treatment to consider for breast masses in such patients. When considering systemic therapy, untreated patients must receive bone marrow transplantation following induced chemotherapy, and patients with extramedullary relapse without bone marrow relapse may undergo strict observation without aggressive systemic treatment. However, when bone marrow relapse occurs, patients must receive systemic chemotherapy and/or a second bone marrow transplantation (9). Case 2 of the current report was diagnosed with GVHD and presented with GS of the breast as the initial symptom following allogeneic bone marrow transplantation. This case is the first such case to be reported and therefore, there were no successful treatments to refer to. Patients diagnosed with extramedullary relapse concomitant with GVHD commonly exhibit liver and renal dysfunction, which reduces the chance of receiving aggressive chemotherapies or a second bone transplantation.

The prevalence of GS of the breast in AML is rare, and a lumpectomy may achieve satisfactory local control and be an alternative treatment for solitary unilateral nodules in the breast.
